# First-in-Man Rapid, Ultra–high-resolution Mapping of the Outflow Tracts Using the Advisor™ HD Grid Catheter

**DOI:** 10.19102/icrm.2021.120107S

**Published:** 2021-01-15

**Authors:** Robert D. Anderson, Geoffrey Lee, Timothy Campbell, Joshua Hawson, Stuart P. Thomas, Saurabh Kumar

**Affiliations:** ^1^Department of Cardiology, Royal Melbourne Hospital, Faculty of Medicine, Dentistry, and Health Science, University of Melbourne, Melbourne, Victoria, Australia; ^2^Department of Cardiology, Westmead Hospital, Westmead New South Wales, Australia; ^3^Westmead Applied Research Centre, University of Sydney, New South Wales, Australia

**Keywords:** Catheter ablation, premature ventricular complex, right ventricular outflow tract, high-density grid mapping

A 65-year-old man without structural heart disease was referred for catheter ablation of highly symptomatic bigeminal, monomorphic premature ventricular complexes (PVCs) believed to be originating from the left ventricular outflow tract (LVOT) (**Figure 1**, panel A). High-density activation mapping was performed using the EnSite™ NavX™ mapping system. PVCs were 15 ms pre-QRS (**Figure 1**, panel A, left column) in the distal coronary sinus and proximal anterior interventricular vein as mapped using a decapolar mapping catheter (Inquiry™). A novel paddle-shaped, high-resolution multipolar catheter (Advisor™ HD Grid), maneuvered to the coronary cusps via a retrograde aortic approach, demonstrated earliest activation in the aortic cusps at the right coronary cusp, 19 ms pre-QRS (**Figure 1**, panel A, middle column and panel D). The septal/free wall of the RVOT demonstrated activation 32 ms pre-QRS (**Figure 1**, panel A, right column; panel B; and panel E). Ablation was performed to the earliest RVOT activation site with immediate cessation of ectopy and consolidative lesions applied (**Figure 1**, panels C and F). Further consolidative lesions were also applied in the right coronary cusp at its earliest site. No immediate complications were recorded and the patient showed no recurrence at six weeks of follow-up. Activation mapping included 350 mapping points collected within 218 seconds of mapping time.

The novel Advisor™ HD Grid catheter is a high-density, 7-French mapping catheter with a paddle configuration with 18 1-mm-wide electrodes arranged in a 4 × 4 configuration with a 3-mm edge-to-edge separation (**Figure 1**, panel G). To our knowledge, this is the first description of its successful use for rapid, ultra–high-resolution activation mapping in the outflow tracts, allowing for differentiation of site of origin and successful ablation eventually in the septal/free wall RVOT despite ECG morphology suggestive of an LVOT origin. The catheter was safe, easily maneuverable in the cusps, and facilitated high-density electroanatomic definition of the cusps.

## Figures and Tables

**Figure 1:**
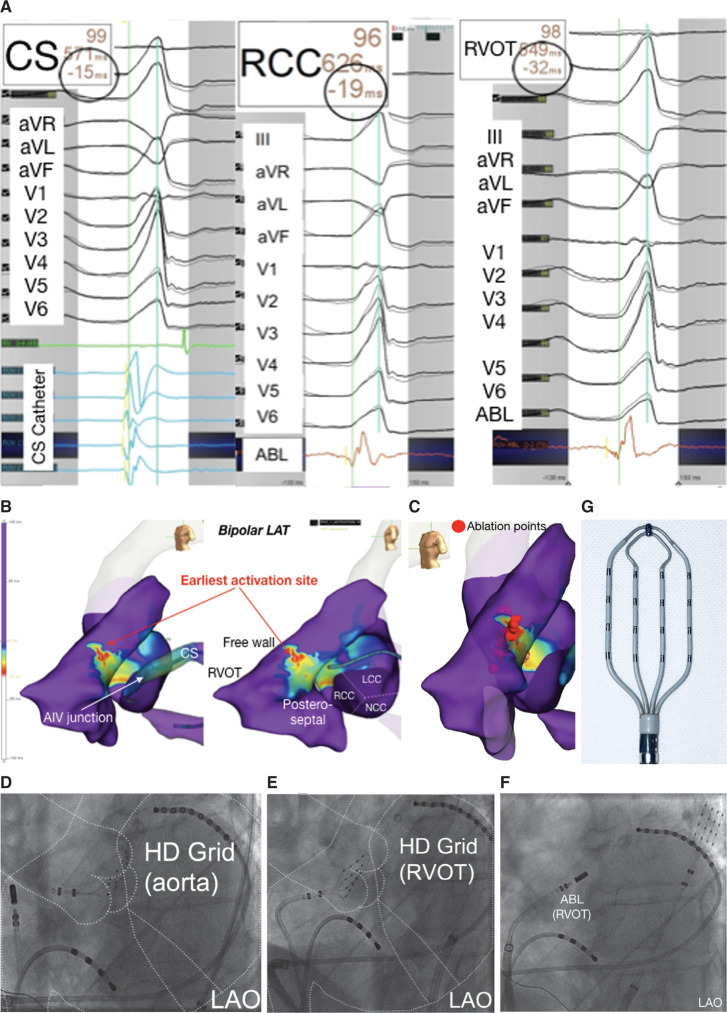
**A:** The clinical PVC and earliest signals from the CS, RCC and RVOT sites. **B:** The LAT map demonstrating the earliest zone, 32 ms pre-QRS, seen in the RVOT. **C:** Ablation lesions. **D–F:** Ablation-associated fluoroscopic images of the Advisor™ HD Grid catheter positioned in the LVOT and RVOT. **G:** Catheter design of the Advisor™ HD Grid catheter.

